# Supplementary material for: What does it take to learn the rules of RNA base pairing? A lot less than you may think
Source: bioRxiv. 2025 Aug 2:2025.07.31.668042. Preprint. [Version 1] doi: 10.1101/2025.07.31.668042 (PMC12324431; doi:10.1101/2025.07.31.668042)
Supplement: Supplement 1 [file media-1.gz › supplemental_material/experiments/g6_optimize_param_S288C_chrV_YER019C-A.rm_v1.200_random/trna1415_annote_2of2.noss.mea/acc_f1_i156.pdf]

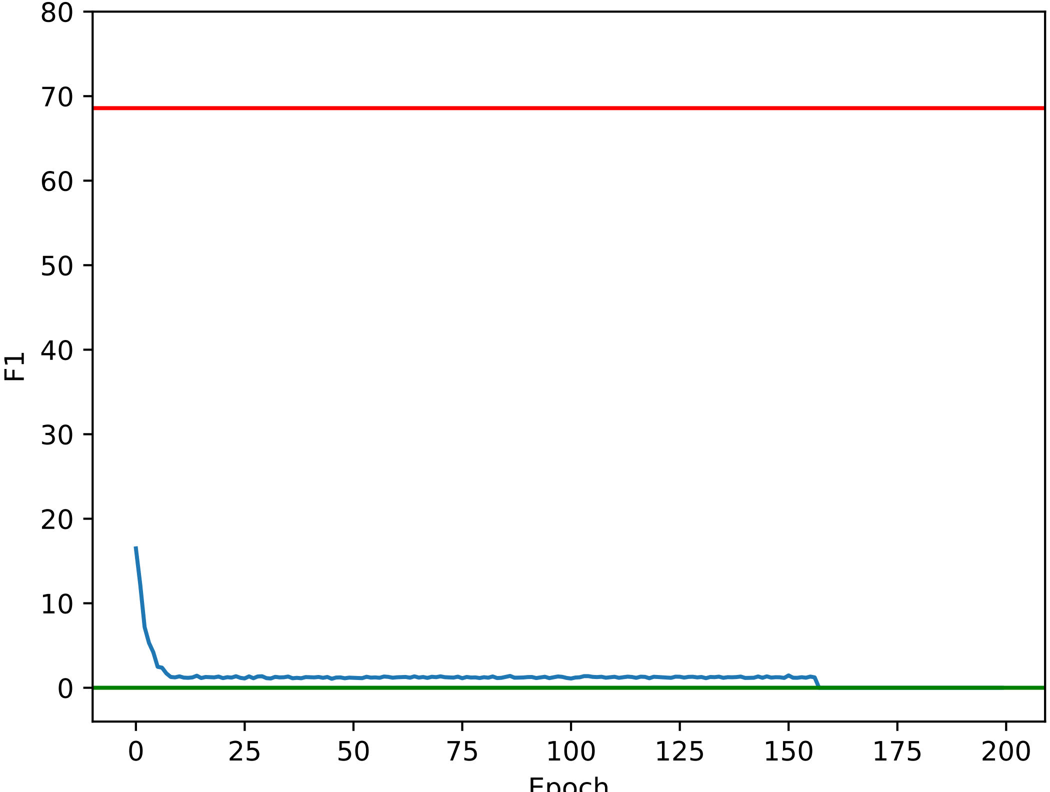

80
70
60
50
40
30
20
10
0
F1
0
25
50
75
100
125
150
175
200
Epoch
